# Assignment constraints in shared transportation services

**DOI:** 10.1007/s10479-020-03522-x

**Published:** 2020-01-25

**Authors:** Margaretha Gansterer, Richard F. Hartl, Sarah Wieser

**Affiliations:** 1 Department for Business Decisions and Analytics, University of Vienna, Oskar-Morgenstern-Platz 1, 1090 Vienna, Austria; 2 Department of Operations, Energy, and Environmental Management, University of Klagenfurt, Universitätsstraße 65-67, 9020 Klagenfurt, Austria

**Keywords:** Logistics, Collaborations, Transportation, ALNS, Central planning

## Abstract

Competitive markets, increased fuel costs, and underutilized vehicle fleets are characteristics that currently define the logistics sector. Given an increasing pressure to act in a manner that is economically and ecologically efficient, mechanisms that help to benefit from idle capacities are on the rise. In the Sharing Economy, collaborative usage is typically organized through platforms that facilitate the exchange of goods or services. Our study examines a collaborative pickup and delivery problem where carriers can exchange customer requests. The aim is to quantify the potential of horizontal collaborations under a centralized framework. An Adaptive Large Neighborhood Search is developed to solve yet unsolved test instances. A computational study confirms the results of past studies which have reported cost savings between 20 and 30%. In addition, the numerical results indicate an even greater potential for settings with a high degree of regional customer overlap. Unfortunately, these high collaborative gains typically come at the cost of an uneven customer distribution, which is known to be one of the main barriers that prevent companies from entering into horizontal collaborations. To generate acceptable solutions for all participants, several constraints are included in the model. The introduction of these constraints to single-vehicle instances, decreases the potential collaborative gain considerably. Surprisingly, this does not happen in more realistic settings of carriers operating multiple vehicles. Overall, the computational study shows that centralized collaborative frameworks have the potential to generate considerable cost savings, while at the same time limiting customer or profit share losses and enabling carriers to keep some of their most valued customers.

## Introduction

Competitive markets, increased fuel costs, underutilized vehicle fleets and stricter customer demands are characteristics that currently define the logistics sector. Due to the increasing competitive pressure, many transport companies have optimized their operations up to an extent where further improvements are not achievable on an individual level (Vanovermeire et al. 2014). On average trucks on European roads are at most half-full, where nearly a quarter of these trucks run empty (IFEU 2008).

The implementation of collaboration networks is an approach that could help tackle this growing lack of efficiency. In the *Sharing Economy* collaborative consumption is typically organized through platforms that facilitate the exchange of goods or services. Past studies have demonstrated that collaboration among competitors can result in considerable cost savings (Gansterer and Hartl 2018b). A collaboration can be described as a partnership between two or more companies to optimize operations by making joint decisions and sharing information, resources or profits (Haider 2014; Simatupang and Sridharan 2002). All participants of a logistics chain can be involved in such a collaboration including suppliers, manufacturers, distributors and customers. If companies at different levels of the chain cooperate, it is referred to as a vertical collaboration (Simatupang and Sridharan 2002; Boros et al. 2008; Yang et al. 2019). For instance, retailers and suppliers might cooperate by sharing information regarding sales and inventory to improve forecasting techniques (Cruijssen et al. 2007c). Another common case of vertical cooperation would be the hiring of third party logistics providers by shippers (Cruijssen et al. 2007b). A horizontal collaboration, on the other hand, involves the cooperation between entities at the same level of a supply chain (Simatupang and Sridharan 2002). This can include joint replenishment between suppliers, or vehicle sharing between carriers (Cruijssen et al. 2007c). The benefits of both approaches can be combined in a lateral collaboration (Simatupang and Sridharan 2002).

Simulation studies on collaborative logistics have been presented by, e.g., Sprenger and Mönch (2012). Based on a real-world setting in the German food industry, the authors show the clear superiority of the cooperative strategy over the non-cooperative performance. The proposed heuristics are tested for the dynamic and stochastic logistics system in a rolling horizon setting using discrete event simulation. A simulation study on request exchange mechanisms for real-world collaborations has been presented by Dahl and Derigs (2011). A multi-agent system that implements a distributed hierarchical algorithm for collaborative transportation planning is assessed by Sprenger and Mönch (2014). Yilmaz and Savasaneril (2012) study the collaboration of small shippers in the presence of uncertainty by simulating a Markov decision process. Quintero-Araujo et al. (2016) discuss the potential benefits of collaborations in supply chains with stochastic demands. A simheuristic approach is used to compare cooperative and non-cooperative scenarios. Quintero-Araujo et al. (2017) quantify potential benefits of horizontal cooperation in urban transportation under uncertainty using a simheuristic approach.

In this study, horizontal collaborations between carriers are examined. The collaboration should enable carriers to exchange customer requests in order to reduce their transportation costs. These kinds of horizontal alliances have been linked to various environmental benefits, including the reduction of $CO_2$ emissions, road congestion and noise pollution. Due to this immense potential, freight-sharing has recently become a widely studied subject in the field of vehicle routing. In reality, however, transport companies have been reluctant to enter horizontal collaborations. Potential participants of collaboration networks have expressed concerns about working with competitors. They fear that instead of profiting from synergy effects, they will lose valued customers and give up potentially damaging information to their competition. A fair workload and profit distribution are considered to be the most important aspects to enable horizontal collaborations in real-world applications. Empirical evidence is provided by Cruijssen et al. (2007b). Based on a large-scale survey on the potential benefits and impediments for horizontal cooperation in Flanders, the authors conclude that in general logistics service providers strongly believe in the potential benefits of horizontal cooperation. However, several barriers do exist. A great majority of respondents agrees that smaller companies in the partnership may lose clients or get pushed out of the market completely. Hence, the authors recommend that potential partners must therefore explicitly take these impediments into account and try to overcome them before cooperation starts. Also Buijs et al. (2018) empirically analyze whether the fear of losing clients to competitors is a barrier for horizontal collaboration among logistics providers. The authors conduct a multi-method approach consisting of observations, in-depth interviews, and a vignette-based experiment. In their empirical results, the authors distinguish between the roles of respondents. They show that in larger companies, where transport planning and outsourcing decisions are taken by different persons, the fear of losing clients to competitors is among the three strongest impediments for horizontal collaborations. These empirical findings emphasize the necessity to numerically examine the impact of allowing participants to keep specific customers or competitive market shares.

Clearly, in transportation collaborations several typical properties of complex systems can be observed: non-linear dependencies, many interacting individuals, competition etc. Even in the deterministic case, we are facing a combinatorial explosion that leads to the fact that only very small instances can be solved to optimality (see Sect. 5). In our numerical study, we are investigating data sets, where the level of complexity is far beyond that. In Sect. 6 we emphasize that analyzing dynamic stochastic behavior of the proposed setting within a simulation environment seems worth investigating.

In Gansterer and Hartl (2018b) it is shown that literature on collaborative vehicle routing follows three streams: (i) centralized, (ii) decentralized auction-based, and (iii) decentralized non-auction-based collaborations. The most effective approach to reallocate these requests would be through a centralized planning system. In this framework, a central authority is in charge of customer allocation. This neutral entity, which is sometimes referred to as a trustee, could for instance be represented by a public authority or an independent online platform. Given complete information, the authority solves an optimization problem in order to maximize the total profit of all participants. While this method could achieve the highest potential cost savings, it involves sharing all relevant information with a third party - something which many companies will be reluctant to do. Additionally, carriers fear being fully exposed to the collaboration in the sense that they are not able to keep certain customers out of the coalition.

As an alternative, decentralized planning systems are proposed, assuming that a fully informed central authority is not required. Instead, a mechanism is put in place to perform exchanges of customer requests. This mechanism could be conducted in an auction-based framework, where companies submit customer requests into an auction pool. Other participants then have the opportunity to bid on submitted requests that are of most value to them. Complex bidding strategies and profit-sharing mechanisms to avoid strategic behavior have to be developed to ensure a functioning system. While non-auction based decentralized systems are generally less complex, they also provide lower savings. A key challenge for both types, however, is identifying which requests have the potential to improve the total collaboration profit and should therefore be submitted for reallocation. Considering that participants do not want to share sensitive information, this identification can become problematic (Gansterer and Hartl 2016).

Horizontal collaborations already play a key role in aviation and maritime transportation. The overall high investment, maintenance and transportation costs provide a strong incentive for competitors to form partnerships (Cruijssen et al. 2007b; Martin et al. 2018; Vanovermeire et al. 2014).

In the case of the less capital-intense road transportation, companies have been more reluctant to enter horizontal alliances (Vanovermeire et al. 2014). Defryn and Sörensen (2018) show that collaborations can be problematic, in particular, if the interests of the players differ significantly. Thus, the authors propose to include individual objectives into a multi-objective optimization procedure, where cost allocation is taken into account. A solution framework for the integration of coalition objectives and partner objectives is presented in Defryn et al. (2019). However, due to the ecological and environmental benefits that are associated with these collaborations, public authorities have a keen interest in increasing cooperation in this sector. This is especially the case for city logistics, where freight transport can contribute between 16 and 50% of transportation emissions (Muñoz-Villamizar et al. 2015). For this reason, public authorities have been funding projects to research potential benefits, construct corresponding platforms and provide additional incentives.

In this respect, the city of Zurich funded a project to develop an online platform facilitating the collaboration between different transport companies. The main idea of the project overseen by Schmelzer et al. (2016) was to link transport companies to a small number of carriers that would carry out last-mile deliveries within the city. A case study consisting of 33 transport companies found that the total distance traveled could be reduced by 32% and estimated cost savings of 18%. Two other projects to study and encourage horizontal collaboration in logistics were launched by the European Union as part of the Horizon 2020 program. The European Commission’s project U-TURN (2018) aims to minimize the carbon footprint and reduce urban delivery costs mainly through promoting shared distribution systems for producers, retailers and distributors. Additionally, project NextTrust (2018) involves conducting over 30 pilots to identify overlapping vehicle movements, less than full vehicles and underutilized transport fleets. The project aims to reduce deliveries and greenhouse gases as well as increase load factors through the identification and elimination of barriers to collaboration. As part of their eCommerce pilot series, a project in Germany is developing a platform to generate a collaborative network for last-mile deliveries. Fleet operators can submit vacant capacities and the system will connect them to an optimized tour, improving delivery times, leading to successful delivery rates and in turn enhancing customer service (NextTrust 2018).

Preliminary results already suggest that over 70% of transport companies would be willing to cooperate with competitors, provided that a neutral trustee oversees the collaboration.

As in empirical studies by Cruijssen et al. (2007b) and Buijs et al. (2018) it is argued that the loss of customers and the fear of being forced out of the market are among the main impediments to horizontal collaborations in practice, the aim of this study is to further extend existing research on centralized collaborations by examining the potential of horizontal collaborations under different restrictions. In this context, we suggest that certain constraints, referred to as assignment constraints, can be imposed to set up acceptable freight-sharing frameworks among carriers. These constraints relate to (i) specific sets of customer carriers do not want to share, (ii) minimum number of customers, and (iii) minimum post-collaboration profits achieved by the carriers. All of them should enable acceptable conditions for carriers to enter horizontal alliances.

It has been shown in Gansterer et al. (2018c) that, in the case of a single vehicle per carrier, these constraints eliminate possible benefits. We extend this research by applying the constraints to multi-vehicle instances. By doing so we can show that in these—more realistic—instances, the additional constraints come at very low cost.

Our study has several contributions:

- we introduce the collaborative multi-depot vehicle routing problem with pickups and deliveries (MDVRPPD).
- a metaheuristic solution method is developed to solve the problem under a centralized authority with and without assignment constraints.
- the proposed solution method is applied to an extensive set of test instances with different characteristics to quantify the potential of carrier collaborations.
- the cost of specific assignment constraints is analyzed in order to assess the remaining benefits of acceptable collaborative solutions.

The remainder of the paper is organized as follows. In Sect. 2 we give a literature review. The problem is introduced and mathematically formulated in Sect. 3. The proposed solution approach is presented in Sect. 4, while the computational study and a deep analysis is given in Sect. 5. Conclusions and further research are summed up in Sect. 6.

## Literature review

Krajewska and Kopfer (2006) and Cruijssen et al. (2007c) were the first to examine the potential benefits of collaborative vehicle routing. Empirical studies were conducted by, e.g., Cruijssen et al. (2007b), Lydeka and Adomavičius (2007), Pateman et al. (2016), to assess the drivers and reservations of companies in the relation to enter horizontal logistics partnerships. Various real-world and hypothetical cases have been examined to determine potential collaboration gains. According to studies conducted by Cruijssen et al. (2007a), Muñoz-Villamizar et al. (2015), Chinh et al. (2016), centralized planning has the potential to improve profits by around 20–30%. Fernández et al. (2018) assess the collaborative gain of a shared customer vehicle routing problem (VRP). They observe savings between 6 and 25%, depending on the geographical distribution of customers.

Studies regarding decentralized planning systems focus mostly on establishing efficient frameworks, especially for complex auction-based systems (Gansterer and Hartl 2018a; Berger and Bierwirth 2010; Dai and Chen 2011; Krajewska and Kopfer 2006).

The challenge of a fair cost or profit allocation is addressed by Hezarkhani et al. (2016), Liu et al. (2010), Vanovermeire et al. (2014), among others. Gansterer et al. (2018b) elaborate on desirable game theoretical properties in auction-based transport collaborations.

Recently, several contributions to the related field of cooperative game theory have been published. Lai et al. (2019), for instance, present an ascending auction for freight forwarder collaboration in capacity sharing. The proposed mechanism iteratively expands the set of bundles and approximates the revenue loss to search optimal allocations. Truthful, budget-balanced bundle double auctions for carrier collaboration are researched by Xu et al. (2016). A game theoretic analysis of horizontal carrier coordination with revenue sharing in E-commerce logistics is presented by Zhang et al. (2019). Seminal work on the *Traveling Salesman Game* and the *Vehicle Routing Game* has been published by Engevall et al. (1998), Göthe-Lundgren et al. (1996) and Engevall et al. (2004). It should be noted that these studies aim at finding core solutions and cost allocation mechanisms, where sub-coalitions of participants are evaluated. In our study, we are solving the ${{\mathcal {N}}}{{\mathcal {P}}}$-hard pickup and delivery problem of the grand coalition and elaborate on the cost of giving the participants flexibility in keeping customers of competitive market shares. To the best of our knowledge, this aspect cannot be answered by the vehicle routing game. The interested reader is also referred to Peleg and Sudhölter (2007), where basics of cooperative game are systematically discussed.

Kimms and Kozeletskyi (2016a) study core-based allocations in the cooperative traveling salesman problem, while Kimms and Kozeletskyi (2016b) use Shapley value-based cost allocation in the cooperative traveling salesman problem under rolling horizon planning.

Environmental aspects are considered by Ballot and Fontane (2010), Muñoz-Villamizar et al. (2015), Pérez-Bernabeu et al. (2015), Schulte et al. (2017). In addition to cost savings of 25%, Muñoz-Villamizar et al. (2015) observe a 9% reduction in the number of routes and a 10% increase in vehicle utilization—factors which can in turn reduce emissions and congestion. Ballot and Fontane (2010) assess a 25% reduction of gas emissions while studying collaborations in French retail chains.

Collaborative VRP are often solved with local search based metaheuristics (Defryn et al. 2016; Pérez-Bernabeu et al. 2015; Sanchez et al. 2016). Berger and Bierwirth (2010) introduce the collaborative pickup and delivery problem, which they solve with two decentralized approaches. Gansterer et al. (2018a) extend the single vehicle case by including workload constraints. They use different exact solution methods including Benders decomposition and column generation to solve the problem under a centralized framework. They conclude that Benders decomposition outperforms the other methods in a setting with fewer constraints, whereas column generation works better if constraints regarding workload distribution are introduced. The single vehicle case is solved heuristically and further analyzed in Gansterer et al. (2018c). It is shown that assignment constraints for single vehicle collaborations, have a detrimental effect on collaboration gains. However, none of these studies consider the more realistic multi-vehicle case.

For more detailed information on studies in the field of collaborative logistics, consult Gansterer and Hartl (2018b) for a general overview of collaborative vehicle routing, Cleophas et al. (2018) for a focus on urban transportation and Guajardo and Rönnqvist (2016) for the development of cost allocation mechanisms.

The vehicle routing problem was first introduced by Dantzig and Ramser (1959). To this day, it remains one of the most widely studied problems in the field of combinatorial optimization (Kritikos and Ioannou 2010). Given a fleet of vehicles and a set of transportation requests, the task is to determine the optimal set of routes to fulfill these requests while respecting given constraints (Irnich et al. 2014). If precedence constraints are imposed, these determine the sequence in which customers can be served within a route. This is especially of importance in the case of pickup and delivery problems (PDP) (Toth and Vigo 2002). PDP can be seen as an extension of the classical VRP (Berbeglia et al. 2007; Parragh et al. 2008). Requests are associated with pickup and delivery locations, where the loading and unloading of goods takes place. Parragh et al. (2008) distinguish between two main classes of PDP: The VRP with backhauls (VRPB) and the VRP with pickups and deliveries (VRPPD).

In recent years equity and fairness aspects have been gaining recognition in both real-world applications as well as theoretical studies in the field of VRP. Balanced resource utilization and a fair workload distribution have been found to provide non-monetary benefits, such as employee satisfaction, increased customer service and flexible resource availability (Matl et al. 2019).

As a result these aspects have been included in vehicle routing models (Bektaş 2013; Huang et al. 2012; Jozefowiez et al. 2008, 2009; Kritikos and Ioannou 2010). This is done either with the help of an objective function or by introducing additional workload constraints. The workload of a tour can be defined as the number of customers visited, the total delivered load or as the distance or time duration (Jozefowiez et al. 2008). In the context of horizontal collaborations, the inclusion of fairness or continuity aspects could help overcome some of the barriers to entry by ensuring a fair workload, cost or profit distribution among participants (Cruijssen et al. 2007b; Buijs et al. 2018). However, the cost of such constraints in the field of MDVRPPD-based collaborations has not been assessed so far. This is the main objective of our study. A classification and available solution methods are surveyed in Dragomir et al. (2018). To the best of our knowledge, collaboration in MDVRPPD with one-to-one, i.e. paired, pickup and deliveries (Berbeglia et al. 2007) has not been researched so far. We want to close this research gap, since these types of problems are considered to be most important in the field of collaborative vehicle routing (Archetti et al. 2014; Gansterer and Hartl 2018b). In the small parcel delivery industry, for instance, idle capacities can easily be shared among collaboration partners.

## Problem formulation

The aim of this study is to assess the potentials of horizontal collaborations among carriers by sharing customer requests. A centralized authority will determine the optimal distribution of customers among participants in order to minimize overall transportation costs.

The underlying problem is a multi-depot PDP as an extension of the classical PDP. Each depot belongs to and therefore represents one carrier. In the following, we refer to depots and carriers synonymously. Each carrier is associated with a number of paired requests consisting of a pickup and delivery point, which will be referred to as the initial customer distribution. Additionally, each carrier is equipped with a certain number of vehicles, starting from and returning to their depot. Each vehicle is only capable of performing one tour. The terms tour and vehicle are therefore used interchangeably. Due to the context of the PDP, a delivery point has to be served after its associated pickup point by the same vehicle. It is known that an uneven cost and profit distribution, as well as the fear of losing customers and market share, are some of the main barriers that prevent companies from entering horizontal collaborations. In order to circumvent this issue, continuity aspects represented by assignment constraints will be included in the model.

Both the single and the multi-vehicle case are examined in our study. In the former case, i.e. the multi-depot traveling salesman problem with pickups and deliveries (MDTSPPD), each carrier only has access to one vehicle with unlimited capacity. For the multi-vehicle case, i.e. the multi-depot vehicle routing problem with pickups and deliveries (MDVRPPD), we include duration and capacity constraints for each vehicle.

The following mathematical model is based on the formulations presented in Dragomir et al. (2018). For the MDTSPPD, the number of vehicles per carrier ($k_l$) is set to one for all carriers. Corresponding capacity (*Q*) and duration (*T*) constraints are set to infinity. A specific formulation for the single-vehicle case can be found in Gansterer et al. (2018a), while the following model addresses the multi-vehicle case. Note that, without assignment constraints, the formulation is equal to a non-collaborative multi-depot VRP (Dragomir et al. 2018).

*R*: Set of customer requests, $R=\{1, \ldots ,n\}$
*P*: Set of pickup nodes, $P=\{1, \ldots ,n\}$
*D*: Set of delivery nodes, $D=\{n+1, \ldots ,2n\}$
*L*: Set of depots (i.e. carriers), $L = \{2n+1, \ldots ,2n+m\}$
*N*: Set of pickup and delivery nodes, $N = P \cup D$
$$N_l$$
Set of all pickup nodes initially assigned to depot *l*, $N_l \subset N$
*V*: Set of all nodes, $V = N \cup L$
$$K_l$$
Set of vehicles at depot *l*, $K_l = \{1, \ldots ,k_l\}$
*K*: Set of all vehicles, $K = \cup _{l\in L}K_l$
$$c_{ij}$$
Cost of traveling from node *i* to node *j*
$$q_i$$
Load of node $$i = \left\{ \begin{array}{rl}+q_i, &{} \forall i\in P\\ -q_i, &{} \forall i\in D\\ 0, &{} \hbox {otherwise} \end{array}\right. $$
*Q*: Load capacity of each vehicle
*T*: Maximum tour duration for each vehicle
$$x_{ijk}$$
= $$\left\{ \begin{array}{rl}1,&{} \, \hbox {if vehicle} \, k \, \hbox {travels directly from node} \, i \, \hbox {to node} \, j \\ 0,&{} \hbox {otherwise} \end{array}\right. $$
$$S_{ik}$$
= Loading amount of vehicle *k* at node *i*
$$t_{ik}$$
= Fulfillment time at node *i* on vehicle *k*

1 $$\begin{aligned}&\displaystyle minimize \sum _{i \in V}\sum _{j \in V}\sum _{k \in K} c_{ij}x_{ijk} \end{aligned}$$

2 $$\begin{aligned}&\displaystyle \sum _{i \in V}\sum _{k \in K} x_{ijk} = 1 \quad \forall j \in N \end{aligned}$$

3 $$\begin{aligned}&\displaystyle \sum _{i \in V} x_{ihk} - \sum _{j \in V} x_{hik} = 0 \quad \forall k \in K, h \in N, \end{aligned}$$

4 $$\begin{aligned}&\displaystyle \sum _{i \in V} x_{ilk} = \sum _{j \in N} x_{ljk} \le 1 \quad \forall l \in L, k \in K_l \end{aligned}$$

5 $$\begin{aligned}&\displaystyle \sum _{j \in N} x_{ijk} - \sum _{j \in N} x_{i+n,j,k} = 0 \quad \forall i \in R, k \in K \end{aligned}$$

6 $$\begin{aligned}&\displaystyle t_{jk} \le t_{ik} + c_{ij} + M(1-x_{ijk})\quad \forall i \in V, j \in V, k \in K \end{aligned}$$

7 $$\begin{aligned}&\displaystyle t_{jk} \ge t_{ik} + c_{ij} - M(1-x_{ijk})\quad \forall i \in V, j \in V, k \in K \end{aligned}$$

8 $$\begin{aligned}&\displaystyle t_{ik} \le t_{i+n,k} \quad \forall i \in R, k \in K \end{aligned}$$

9 $$\begin{aligned}&\displaystyle S_{jk} \le S_{ik} + q_i + M(1-x_{ijk})\quad \forall i \in V, j \in V, k \in K \end{aligned}$$

10 $$\begin{aligned}&\displaystyle S_{jk} \ge S_{ik} + q_i - M(1-x_{ijk})\quad \forall i \in V, j \in V, k \in K \end{aligned}$$

11 $$\begin{aligned}&\displaystyle 0 \le S_{ik} + q_i \le Q \quad \forall i \in V, k \in K \end{aligned}$$

12 $$\begin{aligned}&\displaystyle \sum _{i \in V}\sum _{j \in V} c_{ij}x_{ijk} \le T \quad \forall k \in K \end{aligned}$$

13 $$\begin{aligned}&\displaystyle \sum _{i \in S}\sum _{j \in S} x_{ijk} \le |S|-1 \quad \forall k \in K, S\subseteq N, |S| \ge 2 \end{aligned}$$

14 $$\begin{aligned}&\displaystyle x_{ijk} \in \{0,1\} \quad \forall i,j \in V, k \in K \end{aligned}$$

15 $$\begin{aligned}&\displaystyle t_{ik} \in {{\mathbb {Z}}} \quad \forall i \in V, k \in K \end{aligned}$$

16 $$\begin{aligned}&\displaystyle S_{ik} \in {{\mathbb {N}}} \quad \forall i \in V, k \in K \end{aligned}$$

The objective function (1) minimizes overall travel costs. Equation (2) ensures that each pickup and delivery point is visited exactly once. If a request is visited by a vehicle, the same vehicle has to leave that node as well (3). Additionally, pickup and delivery points have to be visited within the same tour (5). Each vehicle has to start from and return to their assigned depot and is restricted to one trip (4). Constraints (6) and (7) compute the travel time at each node, which is used to ensure precedence constraints between pickup and delivery points (8). The load at each node is determined by (9) and (10) and constrained by (11). Duration constraints are met by (12) and constraint (13) prevents the use of subtours. Finally, constraints (14) to (16) define the decision variables.

The mathematical model minimizes the costs of all vehicles, without considering aspects of equal distribution aspects. Solutions of MDTSPPD as well as MDVRPPD may lead to unevenly distributed solutions where all customers are assigned to only one carrier. In a collaborative setting, this is clearly not desirable and will scare off potential participants. Companies may be more likely to enter collaborations if they can, e.g., keep some of their current customers (Cruijssen et al. 2007b; Buijs et al. 2018). This is a reasonable request, given that many companies have valuable long-term customers that they do not want to lose.

We examine the potential of collaborative solutions by considering three different settings for the MDVRPPD:

- each carrier wants to keep a minimum amount (a subset) of his initial customers (*A*),
- each carrier wants to keep a certain percentage of customers, no matter whether these customers initially were served by this carrier or not (*B*),
- each carrier wants to keep a minimum profit with respect to the status quo, resulting in an upper bound on profit losses (*C*).

We add the following parameters:

$$\delta _i$$
Revenue when serving node *i*
$${\varOmega }_l$$
Minimum workload (number of customers) at depot (i.e. carrier) *l*
$${\varOmega }_l^p$$
Minimum profit at depot (i.e. carrier) *l*

The mathematical model is extended by the following constraints (17), (18), and (19) for restrictions A and B, respectively:

17 $$\begin{aligned}&\displaystyle \sum _{i \in V}\sum _{j \in N_l}\sum _{k \in K_l} x_{ijk} \ge {\varOmega }_l \quad \forall l \in L \end{aligned}$$

18 $$\begin{aligned}&\displaystyle \sum _{i \in V}\sum _{j \in P}\sum _{k \in K_l} x_{ijk} \ge {\varOmega }_l \quad \forall l \in L \end{aligned}$$

19 $$\begin{aligned}&\displaystyle \sum _{i \in V}\sum _{j \in V}\sum _{k \in K_l} \delta _ix_{ijk}-c_{ij}x_{ijk} \ge {\varOmega }_l^p \quad \forall l \in L \end{aligned}$$

For setting A, (17) has to hold for the set of customers initially being assigned to depot *l*, i.e. $N_l$, while for setting B, all customers are considered. This is formulated in (18). For setting C, which is given in (19), we have to include each customer’s revenue and subtract the total travel cost of the carrier running depot *l*. For each setting (A,B,C), ${\varOmega }_l$ or ${\varOmega }_l^p$ are predetermined parameters that depend on the carriers’ initial situations.

Note that we can easily guarantee that each carrier is better off by assuming the profit-based ${\varOmega }_l^p$ to be more than 100% of the initial profit of carrier *l*. However, even if ${\varOmega }_l$ is less than that, individual losses can be avoided, since the overall collaboration profit can be used to compensate participants for potential losses. The exceeding total profit (after compensation payments) can be distributed, making use of any profit sharing method. It should be noted that not all available profit sharing methods guarantee individual rationality. Guajardo and Rönnqvist (2016) provide an extensive survey on these approaches. A new method that distributes profits based on individual contributions is proposed by Gansterer et al. (2019).

## Solution approach

Vehicle routing problems are typically NP-hard. Exact solution methods for the MDVRPPD are therefore limited to smaller instances. Readers interested in exact solutions approaches are referred to Gansterer et al. (2018a), where different methods for the single-vehicle case are compared. It is shown that, depending on the method, workload constraints can help the solution procedure. However, to extend the problem to larger instances, a metaheuristic approach is needed. The ALNS was chosen because it has been widely used in the field of PDP problems and has been proven to find good solutions in a reasonable time (Ghilas et al. 2016; Li et al. 2016; Masson et al. 2013). In fact, the first ALNS framework was developed specifically for a PDP problem by Ropke and Pisinger (2006). We extend an ALNS proposed in Pisinger and Ropke (2007) by new operators, which are tailored to the imposed assignment constraints.

Information on the solution framework and additional notations are given in Sect. 4.1. The main removal and insertion operators are explained in Sect. 4.2. Section 4.3 provides an overview of the ALNS framework and a detailed explanation of its individual components.

### Solution framework and notations

The ALNS will be applied to five cases. First, the costs of a non-collaborative situation constrained by the initial customer distribution are computed. This means each carrier faces a classical PDP with only one depot and one (MDTSPPD) or multiple (MDVRPPD) vehicles at their disposal. Additionally, the collaborative solutions are determined by imposing no assignment constraints and by imposing constraints A–C.

In the following, notations and terms used are defined. The term *position* will encompass information on the tour a request is part of, as well as the position of both pickup and delivery node in that tour. A solution *S* encompasses $\sum _{l \in L}{k_l}$ tours. These individual tours will be denoted by $S_t$, $\forall t \in K$. Each solution *S* is associated with its objective value *f*(*S*). The set of removal operators is denoted by *RO* and the set of insertion operators by *IO*.

### Operators

The calculation of saving and insertion costs (Sect. 4.2.1) is based on Renaud et al. (2000) and extended to the case of multiple depots. The operators presented in Sects. 4.2.2 and 4.2.3 follow Ropke and Pisinger (2006).

#### Cost savings and insertion costs

The essence of the ALNS lies in destroying a solution by removing requests and then repairing that solution through reinserting removed requests. By definition, the distance between two nodes can never be negative. The removal of a request will therefore always be associated with cost savings and the insertion of a request with insertion costs. Considering that the majority of operators select requests based on these values, their computation will be explained in more detail. In both cases, two scenarios have to be distinguished: If a pickup node *i* and its corresponding delivery node $i+n$ are currently located or should be inserted directly next to each other, it is referred to as scenario 1. If instead $i+n$ is currently located or should be inserted in some other position after *i*, it is referred to as scenario 2.

Let *k* be the the direct predecessor of *i* and *l* the direct successor of $i+n$. Both the savings of removing and the costs of inserting request *i* in scenario 1 are then calculated by function (20).

20 $$\begin{aligned} c_{k,i} + c_{i,i+n} + c_{i+n,l} - c_{k,l} \end{aligned}$$

Let *r* be the direct successor of *i* and *s* the direct predecessor of $i+n$. The savings and insertion costs in scenario 2 are then calculated by function (21).

21 $$\begin{aligned} c_{k,i} + c_{i,r} - c_{k,r} + c_{s,i+n} + c_{i+n,l} - c_{s,l} \end{aligned}$$

**Fig. 1:**
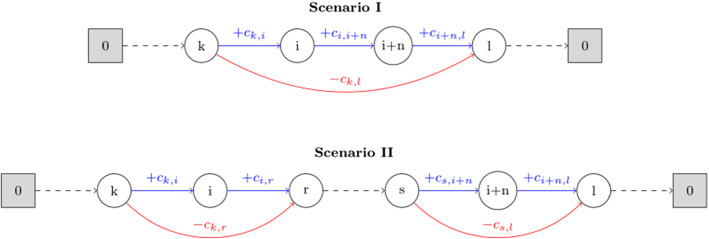
Illustration of the calculation of insertion costs and cost savings for scenarios 1 and 2

Both scenarios are depicted in Fig. 1. In the case of removal operators, blue arcs represent the connections that have to be removed and red arcs the connections that have to be included to reconnect the tour. In the case of insertion operators, blue arcs represent the connections that are to be inserted, and red arcs the abundant connections that have to to be removed. In each scenario, the depot is represented by the node zero.

#### Removal operators

Three removal heuristics are implemented to destroy an existing solution by removing a predefined number of *q* requests ($|RO| = 3$). They include random removal, worst removal and related removal. All of them take a solution *S* with *n* requests as their input and return a solution with ($n-q$) requests.

*Random Removal (R1)*: The random removal heuristic is the simplest operator, as it merely picks *q* requests at random and removes them. Its function relies mainly on diversifying the search and thus helps to escape local optima.

*Worst Removal (R2)*: The worst removal heuristic first calculates the cost savings of all requests that are currently part of the solution. The requests are then sorted in a list with descending order according to their savings. Instead of always choosing the request with the highest savings, Ropke and Pisinger (2006) propose including some level of randomness. They introduce a parameter $p_{worst} \ge 1$ and a random number $y\in [0,1)$. Depending on the parameter $p_{worst}$, requests at the beginning of the list have a higher chance of being selected. If $p_{worst}$ is set equal to one, complete randomness is ensured. A higher value of $p_{worst}$ increases the likelihood that the request with the highest savings is selected.

*Related Removal (R3)*: The related removal heuristic was introduced by Shaw (1997). It tries to determine requests that are somehow related to each other and should therefore be removed together. In the context of the PDP with time windows, Ropke and Pisinger (2006) define this relatedness regarding the distance between two requests and their difference in service time, load and vehicle compatibility.

Considering that there are no time window or compatibility constraints in the problems examined in this study, relatedness refers only to distance and load in the case of the MDVRPPD. In the context of the MDTSPPD, the relatedness measure encompasses only the distance term, as no load capacities are considered.

*Distance similarity* $D_{i,j}$ is calculated by adding the distance between the pickup points and the delivery points of two requests *i* and *j* and is weighted by the factor $\alpha$ (22).

22 $$\begin{aligned} D_{i,j} = c_{i,j} + c_{i+n,j+n} \end{aligned}$$

*Load similarity* $L_{i,j}$ is defined as the absolute difference between the load of *i* and the load of *j* and is weighted by factor $\beta$.

The *relatedness measure* $R_{i,j}$ is then calculated by adding up the weighted similarity terms according to Eq. (23). For the MDTSPPD, $\beta$ is simply set to zero.

23 $$\begin{aligned} R_{i,j} = D_{i,j}*\alpha + L_{i,j}*\beta Requests are removed with regard to this relatedness measure.\nonumber \\ \end{aligned}$$

A request currently part of the solution is chosen at random and marked as the seed node *r*. The relatedness measures between *r* and all other requests in the solution are calculated. The requests are then sorted in a list with descending order according to their relatedness measure. Similarly to R2, *q* requests are removed, where requests with a higher relatedness measure have a greater chance of being selected.

#### Insertion operators

Six insertion operators are used in total to repair a solution by reinserting the removed requests ($|IO| = 6$). They include a greedy insertion, two versions of a regret insertion and two modifications to promote assignment constraints. All of the insertion operators used can be described as parallel construction heuristics. The insertion operators take a solution *S* with less than *n* requests and return a solution with *n* requests.

*Greedy Insertion (I1, I2)*: The greedy insertion operator—also referred to as *best* or *basic greedy* insertion (Masson et al. 2013; Ropke and Pisinger 2006)—is a simple and fast heuristic. At each iteration, the minimum insertion costs of each request currently not part of the solution are determined. Functions (20) and (21) are used to calculate the insertion costs of a request for all feasible positions in the solution. The position which yields the smallest insertion cost is defined as the best position for request *i* and the corresponding cost as $minIC_i$. Among all requests $i \notin S$, the one with the smallest $minIC_i$ is inserted at its best position. This process is continued until all requests are included in the solution. Ropke and Pisinger (2006) propose introducing a noise parameter to diversify the search. Following the formulation from Ghilas et al. (2016), a noise parameter $\mu$ and a random number $\epsilon \in [-1,1]$ are used to perpetuate the insertion cost. The following value is then added, where ${\overline{d}}$ is defined as the largest distance between nodes.

24 $$\begin{aligned} InsertionCost_{randomized} = InsertionCost + {\overline{d}}*\mu *\epsilon \end{aligned}$$

Insertion operator I1 is defined as a greedy insertion heuristic without noise and insertion operator I2 with noise.

*Regret Insertion (I3, I4)*: Instead of only taking into account the best insertion position of a node, regret heuristics evaluate the cost of not inserting a node at its best position. At each iteration, a regret value is calculated for all unvisited requests. The request with the highest regret value is selected and inserted into its best position. In I3, this regret value is defined as the difference between inserting a request at its best position and inserting a request at its second best position. In I4, the regret value is defined as the difference between inserting a request in its best position among all tours and inserting the request in its best position in another tour.

*Assignment Insertion (I5, I6)*: To reduce violations of assignment constraints, two assignment operators are included. They are based on operators I1 and I4, respectively. For operators I5 and I6, however, the probability of inserting a request in a tour rises with the fairness violation of the corresponding depot. If no assignment constraints are introduced, the same probability is given to all depots. When using operators I5 and I6, only tours belonging to the selected depot are eligible for insertion, and the insertion cost (I5) or regret value (I6) are modified by adding a violation value to the second term of Eq. (24). A pseudo code for the general framework of the insertion operators is provided in Algorithm 1.
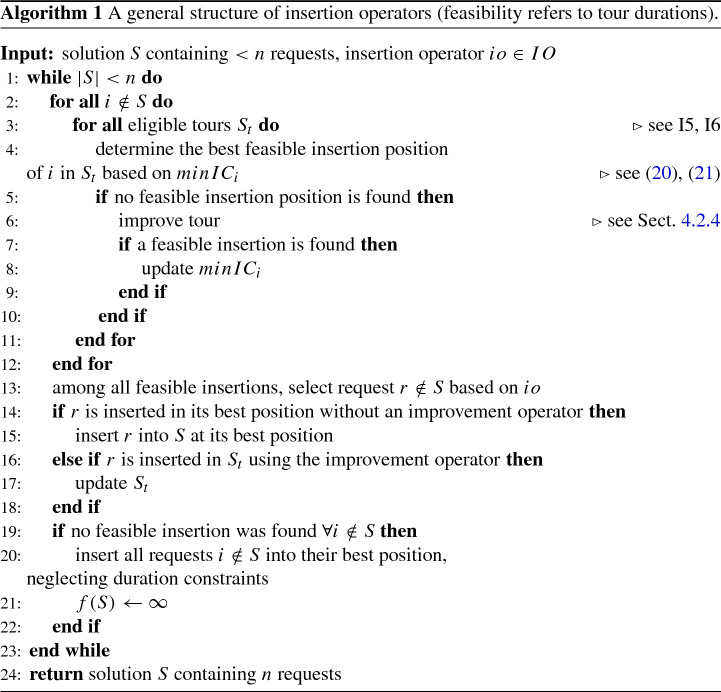


#### Feasibility

**Fig. 2:**
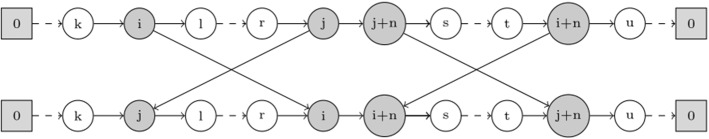
For each exchange of requests *i*, *j*, the swap operator exchanges the position of pickup nodes *i*, *j* and the delivery nodes $i+n$,$j+n$. In doing so, precedence constraints remain fulfilled

All insertion operators presented in Sect. 4.2.3 aim to return a feasible solution in terms of precedence, capacity and duration constraints. The *precedence constraints* are guaranteed by only considering positions following the pickup point in the same tour for the delivery point. Similarly, *capacity restrictions* are ensured by only examining insertion positions which would not lead to a capacity violation. At the very least, this constraint can always be fulfilled by inserting pickup and delivery point of a request directly next to each other at the end or the beginning of a tour. Considering that only a limited number of vehicles is available, the iterative insertion of nodes can lead to cases where tour *duration constraints* are not fulfilled. Due to this issue, an improvement heuristic is performed if the insertion of a request would lead to a duration violation. Specifically, an intra-tour node exchange is applied to the corresponding tour. The swap operator is illustrated in Fig. 2. If, despite this improvement, no feasible insertion position can be found for a request, the solution is marked infeasible and its objective value is set to infinity. It should be noted that without assignment constraints the insertion heuristics will always guarantee feasibility for the MDTSPPD, as there are no duration constraints.

The *assignment constraints*, on the other hand, pose much stronger restrictions to the solution. As it is difficult to find a solution that complies with these constraints, the insertion heuristics themselves do not seek to fulfill them. Instead, a penalty is added to the objective value of a solution if assignment constraints are violated. This is to enforce the algorithm to leave infeasible regions.

Following Kovacs et al. (2014), an artificial objective function $f_a(s')$ is calculated for solutions that violate only the assignment constraints.

25 $$\begin{aligned} f_a(S') = f(S') + \gamma *violationDegree * e^{\frac{it}{\delta }-p_{penalty}} \end{aligned}$$

The penalty is therefore determined by the number of iterations currently performed (*it*), parameters $\delta$ and $p_{penalty}$, and a normalized value (*violationDegree*) indicating the level of assignment constraint violation weighted by parameter $\gamma$. As the number of performed iterations increases so does the penalty, making it less likely to accept infeasible solutions. This way the search process can explore infeasible spaces, while at the same time working towards a feasible solution.

While three different assignment constraint violations are considered, the approach is standardized for all of them. The violation degree per carrier is therefore defined relative to the minimum workload for all assignment constraints (A–C):

26 $$\begin{aligned} violationDegree_t = 1 -\frac{C_{current}}{C_{required}} \end{aligned}$$

The term $C_{required}$ stands for the minimum and $C_{current}$ for the current number of initial customers (A), total customers (B) or profit (C) that has to be held. Since only solutions with assignment constraint violations are considered, the following rule applies: $C_{required} > C_{current}$. The $violationDegree_t$ will therefore always be within the range of (0; 1] for constraints A and B. For constraint C, it may exceed 1, if the current profit is negative. The total *violationDegree* is composed by adding up the violation degrees of all carriers *t*.

### The adaptive large neighborhood search

In the following section, the framework of the ALNS including all relevant elements, will be explained in detail.

#### General framework

First, an initial solution has to be generated on the basis of a constructive heuristic (Sect. 4.3.3). The algorithm is initialized with a best known objective value of infinity and the initial solution *S*. If the initial solution is feasible with respect to all constraints, the best known solution $S_{best}$ is updated. At each iteration a number of requests *q* is chosen at random within the given range $[q_{min}, q_{max}]$. A removal operator $ro \in RO$ and an insertion operator $io \in IO$ are chosen on the basis of a *roulette wheel selection*. The current solution $S'$ is then partially destroyed by removing *q* requests with the removal operator *ro*. Removed requests are reinserted by applying *io* on $S'$. The incumbent solution *S* is updated if the current solution $S'$ fulfills a certain acceptance criterion (Sect. 4.3.2). If the current solution $S'$ is feasible and leads to a lower objective value than the currently known best solution, $S_{best}$ is also updated. If, however, the current solution is not accepted, $S'$ is reset to the incumbent solution *S*. At the end of each iteration, the weights of the operators *io* and *ro* are updated according to their performance. The process continues until some stopping criterion (Sect. 4.3.2) is met.

#### Acceptance and stopping criterion

The stopping criterion is defined as a certain number of iterations (*iter*) to be performed.

Whether or not an incumbent solution is accepted depends on the acceptance criterion. In this study, the linear Threshold Acceptance, introduced by Dueck and Scheuer (1990), is used. According to this concept, a solution $S'$ is accepted if its objective value is not more than *T* percent higher than the objective value of the incumbent solution *S*.

The threshold is initialized with $T_{start}$ and decreases at each iteration by a constant amount until $T_{end}$ is reached (27).

27 $$\begin{aligned} \frac{(T_{start}-T_{end})}{iterations} \end{aligned}$$

Ropke and Pisinger (2006)—along with many other comparable studies (e.g. Azi et al. 2014; Ghilas et al. 2016; Kovacs et al. 2014; Li et al. 2016; Masson et al. 2013)—use a Simulated Annealing approach as the acceptance criterion. In their recent evaluation of acceptance criteria for the ALNS, however, Santini et al. (2017) conclude that Simulated Annealing does not necessarily provide better results and that linear threshold acceptance excels both in simplicity and in the quality of solutions. Infeasible solutions in terms of distance and assignment constraints are rejected until a feasible solution is found. Afterwards they may be accepted as the current solution with an objective value of infinity for distance violations or penalized by (25) for assignment constraint violations. The threshold $T_{start}$ is used until the first feasible solution is found. Only then, the threshold will start to decrease by setting *iterations* to the number of remaining iterations.

#### Initial solution

Initial solutions are constructed with a regret-based insertion heuristic. Since only a certain number of nodes are removed and reinserted at each iteration, it may be difficult for the algorithm to get from a solution with a high violation of assignment constraints to a feasible solution. Therefore, all initial solutions are required to fulfill these constraints. The constructive heuristic is based on insertion heuristic I3. If assignment constraints are not fulfilled, only the tours of violated carriers are eligible for insertion. For constraint C, the finding of a feasible solution cannot be guaranteed by a constructive heuristic. In light of this fact, the non-collaborative solution is used as an initial solution. When solving the MDTSPPD, initial solutions are always feasible. For the MDVRPPD the possibility of a duration violation remains, in which case the objective value of the solution is set to infinity. Considering that infeasible solutions are rejected until a feasible solution has been found, the algorithm will first try to improve the initial solution in order to fulfill the duration constraints before it can explore neighborhoods that may violate assignment constraints.

The main feature that distinguishes the ALNS from the LNS, is the use of various destroy and repair heuristics that are chosen with the help of an adaptive selection procedure.

Initially all removal operators $ro \in RO$ and all insertion operators $io \in IO$ are assigned the same weight $W^{-}_{ro}$ and $W^{+}_{io}$, respectively. The initial value of all weights is set to one. According to Ropke and Pisinger (2006), a roulette wheel selection principle is used on the basis of these weights. The probability of selecting an operator is determined separately for removal and insertion heuristics.

## Computational study and discussion

The numerical experiments aim to quantitatively measure potential benefits of collaborative solutions in comparison to the status quo, as well as the trade-offs when constraints (A–C) are introduced. The ALNS described in Sect. 4 was implemented using different data sets for both the MDTSPPD and the MDVRPPD. The algorithm was coded in C++ and the experiments were carried out single-threaded on an Intel Core i5-7500 processor with 2.70 GHz.

All instances are created by generating equidistant carrier depots with a distance of 200. Customer requests are then randomly added within a radius of 150, 200 and 300 from their carrier’s depot. This results in three different settings O1–O3, which differ in the geographical distribution of customers.

**Fig. 3:**
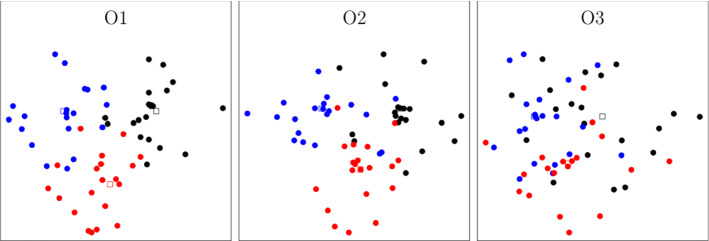
An illustration of the different distribution settings

The corresponding data sets will be referred to as TSP_O1 to TSP_O3 for the single vehicle case and VRP_O1 to VRP_O3 for the multi-vehicle case. The first setting (O1) is characterized by a clustered location of requests around their initial depots. In the third setting (O3), customers are instead distributed more randomly around all depots. This in turn leads to a higher level of competition between the carriers. Figure 3 illustrates the regional differences based on an exemplary instance of the single vehicle case. The distinction goes in line with Berger and Bierwirth (2010), where three different degrees of customer overlap are distinguished.

All data sets are divided into two classes where each carrier initially holds either 10 or 15 customer requests. For each scenario, 20 instances are created, leading to a total of 120 instances for both the single and the multi-vehicle case. All instances include three depots which represent the carriers. This results in a total of 30 and 45 customer requests, respectively. For the MDVRPPD, three vehicles—subject to duration and load constraints—are available at each depot. For the MDTSPPD instances, there is only one vehicle available at each depot, which is not subject to any additional constraints. The cost of traveling between nodes i and j ($$c_{ij}$$) is defined as the Euclidean distance between them. All instances are publicly available.^1^$$^{,}$$^2^

The ALNS contains several parameters which can determine the quality of the solution. The initial parameters were taken from the literature and are for the most part based on Ropke and Pisinger (2006). Sensible parameters such as the reaction factor *r*, the roulette wheel parameters $\sigma _1$, $\sigma _2$, $\sigma _3$, the infeasibility parameters $p_{penalty}$ and $\gamma$, as well as the starting and ending acceptance threshold $T_{start}$ and $T_{end}$ were adapted. The tuning was performed sequentially as proposed by Ropke and Pisinger (2006). One parameter was changed while the others remained constant. Combinations of parameter values were applied to the Berger and Bierwirth (2010) instances and compared to the optimal solutions. The combination that led to the best objective value over three runs was chosen. Additionally, the interval of nodes to be removed [$q_{min}$, $q_{max}$] was extended and the number of iterations was set to 100. The final parameter setting for the ALNS is shown in Table 1.

**Table 1:** Parameter settings for the ALNS

Parameter	Value	Description
*iter*	100	Number of iterations
$q_{min}$	5% of |*R*|	Lower limit of removable requests
$q_{max}$	35% of |*R*|	Upper limit of removable requests
$\sigma _1$	10	Weight adjustment: score for new global best
$\sigma _2$	5	Weight adjustment: score for new better solution
$\sigma _3$	15	Weight adjustment: score for new worse solution
*r*	0.4	Weight adjustment: reaction factor
$p_{worst}$	3	Worst removal: randomization parameter
$p_{related}$	6	Related removal: first parameter
$\alpha$	9	Related removal: second parameter
$\beta$	3	Related removal: third parameter
$\mu$	0.1	Noise parameter
$\delta$	20	Infeasibility: first parameter
$p_{penalty}$	2	Infeasibility: second parameter
$\gamma$	0.1$\cdot {\overline{d}}$	Infeasibility: third parameter
$T_{start}$	8%	First threshold
$T_{end}$	2%	Last threshold

In Table 2 we compare the proposed algorithm against available benchmarks, where assignment constraints are not considered. For the single-vehicle case, optimal results for test instances provided by Berger and Bierwirth (2010) (O1–O3) are available (Gansterer et al. 2018a). In Table 3 we provide a comparison of the proposed ALNS against the available optimal results.

**Table 2:** Comparison of algorithm (without assignment constraints) against best-known solutions (BKS)

Instance	Gap (%) to BKS (%)
O1	3.48
O2	5.2
O3	5.44
VRP_O1	1.56
VRP_O2	$-$0.5
VRP_O3	$-$4.8

We report average percentage gaps. Negative numbers indicate that new BKS could be found

The results show a relatively low average percentage gap of less than 3%. However, it should be noted that optimal results are available for small 1-vehicle instances only. Even with state of the art exact approaches larger instances could not be solved so far (Gansterer et al. 2018a). However, for the multi-vehicle case, we compare against best-known results obtained by several methods including a powerful combinatorial auction approach (Gansterer et al. 2019).

**Table 3:** Comparison of the proposed algorithm against exact solutions for small 1-vehicle instances provided by Berger and Bierwirth (2010) and solved in Gansterer et al. (2018a)

Instance set	Gap (%) to BKS (%)
O1	1.67
O2	5.41
O3	1.17
Average	2.51

We report average percentage gaps

The results show that for two sets of instances (VRP_O2 and VRP_O3) the proposed ALNS finds new best solutions. For single-vehicle instances (O1–O3), where optimal solutions are available, the maximum gap is 5.44%. Overall these results indicate that the performance of the selected solution approach is sufficiently good in order to be adapted for the newly introduced scenarios.

In addition to the ALNS parameters, the assignment constraints have to be defined as well. For constraint A, the setting implemented in Gansterer et al. (2018a) was chosen. Therefore, the requirement that each carrier has to keep at least $\frac{1}{3}$ and $\frac{2}{3}$ of their initial customer base is imposed. For instances with 45 requests in total, the minimum workload is set to 5 and 10 initial customers for each carrier. For instances with 30 requests the minimum workload is set to 3, 4, 6 and 7 initial requests. Then, the weighted average of keeping 3 and 4 customers is computed for $\frac{1}{3}$ and the weighted average of keeping 6 and 7 customers is computed for $\frac{2}{3}$. To examine the difference between keeping specific customers and keeping only a number of customers—regardless of who they were originally assigned to—the same setting is implemented for constraint B as well. Constraint C guarantees a certain profit to potential participants relative to their profits from a non-collaborative solution.

While it is reasonable to assume that carriers may want to keep at least a few of their original customers, a maximum profit loss of $\frac{2}{3}$ does not appear to be a satisfying guarantee. Instead, a stricter minimum workload of 80% and 90% of the initial profit is set for constraint C. An overview of the different workload settings is given in Table 4.

**Table 4:** Constraint settings: The table shows the two minimum workloads that are tested for constraints A–C

	Keep at least		
A:	33.33%,	66.67%	Of the initial customer base
B:	33.33%,	66.67%	Of the initial $\#$ of customers
C:	80.00%,	90.00%	Of the initial profit

First, the potential cost savings of a collaborative solution in comparison to a non-collaborative will be examined. Then, the effect of imposing the three different assignment constraints will be analyzed (A–C).

### Potential collaboration gain

The ALNS is applied to both the collaborative and the non-collaborative setting to assess the potential collaboration gain. Table 5 reports the relative gap between initial and collaborative solution.

**Table 5:** Total collaboration gain: the average gap between initial and collaborative solution without assignment constraints

Instances	n = 30	n = 45	Average	Instances	n = 30	n = 45	Average
TSP_O1	12.53	13.48	13.01	VRP_O1	8.46	13.67	11.07
TSP_O2	24.28	22.09	23.18	VRP_O2	18.12	15.28	16.70
TSP_O3	39.38	35.45	37.42	VRP_O3	25.22	20.00	22.61
Average	25.40	23.67	24.54	Average	17.27	16.32	16.79

All numbers are reported in percentage points

According to the numerical results, collaboration among carriers can lead to cost savings of around 25% (MDTSPPS) and 17% (MDVRPPD) on average. These findings go in line with past studies, where collaborative gains of 20–30% are reported (Chinh et al. 2016; Cruijssen et al. 2007a; Muñoz-Villamizar et al. 2015). The cost savings could even go up to around 35–40% when there is a strong regional overlap of customers, as demonstrated by setting O3. A smaller degree of customer overlap decreases the potential collaborative gain. This becomes especially apparent in setting O1, where customers are located closely around their initial depots. The reassignment to other carriers will therefore often be associated with additional costs. Furthermore, the results suggest that the collaborative gain decreases with the total number of customers. Carriers with many customers have more flexibility to build efficient tours and therefore less incentives to collaborate with others. The additional flexibility seems to decrease with the level of competition and vanishes in setting O1.

This effect could be explained with a high probability to have customers close to the depot of competitors. The potential cost savings of collaborative solutions, however, are considerably smaller for the VRP instances. The average gains of over 20% in the single-vehicle case, can only be achieved in the highly competitive setting O3.

**Fig. 4:**
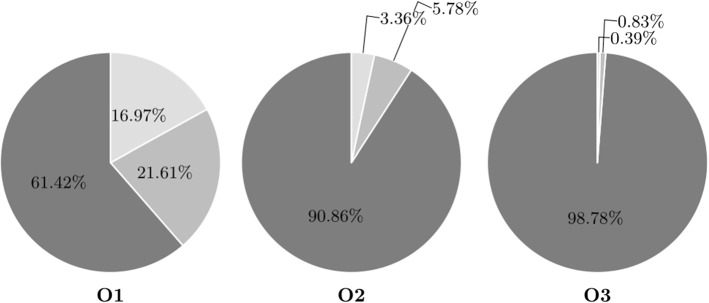
An illustration of the average customer distribution among carriers for settings O1–O3 (MDTSPPD). The carrier with the highest customer share is reflected by a darker color, and the carrier with the lowest customer share by a lighter color

**Fig. 5:**
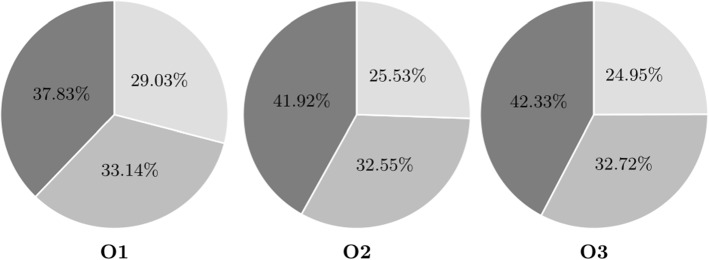
An illustration of the average customer distribution among carriers for settings O1–O3 (MDVRPPD). The carrier with the highest customer share is reflected by a darker color, and the carrier with the lowest customer share by a lighter color

Despite these large potential savings, companies hesitate to enter horizontal collaborations for fear of customer and profit loss. The computational results show that these concerns are not unsubstantiated. Figures 4 and 5 illustrate the average customer distribution among carriers for the different settings.

In instances with a high degree of regional customer overlap, one carrier ends up serving nearly all customers. For the instances with less competition, this effect is reduced. These findings go in line with the significantly smaller collaboration gain reported for setting O1. Even in setting O1, however, customers are unevenly distributed among carriers. This can be explained by the fact that all vehicles have to start from and return to their depot. Even though customers are located around their initial carrier, it might be more efficient to include the requests within an existing tour. This way an additional trip to a depot can be avoided. Once a customer from a competitor is included in a tour, all other customers of that carrier can easily be reached as well. In either case, it is clear that this distribution is not desirable for potential participants.

In contrast to the single-vehicle case, customers are more evenly distributed among carriers. In a setting with less customer overlap (O1), there is in fact not much difference between the workload of the carriers. In more competitive settings (O2, O3), one carrier does hold significantly more customers than the other participants.

Despite some imbalances, no carrier holds a market share of over 50% and even the carrier with the lowest market share is able to serve a quarter of all customers. This is a considerable difference to the single-vehicle case where one carrier ended up serving nearly all customers and can explain the lower collaboration gain. In the multi-vehicle case, only a certain number of customers can be visited within one tour. Once a second route has to be opened, the benefits of avoiding the trip to the depot are eliminated. The remaining customers may then be located closer to competitors, resulting in evenly distributed solutions. Table 6 gives additional insights on the vehicle utilization among carriers.

**Table 6:** Vehicle utilization: The table reports the percentage of instances where each carrier uses at least 1, 2 or 3 vehicles in the collaborative solution

Instances	1	2	3
VRP_O1	100.0	100.0	57.5
VRP_O2	100.0	100.0	50.0
VRP_O3	95.0	92.5	45.0

For settings O1 and O2, every carrier uses at least two vehicles in all observed instances. In setting O3, there are at least some instances where one carrier does not use a single vehicle and therefore also serves no customers.

The findings enforce the need for assignment constraints to generate acceptable solutions for all participants. Introducing these constraints can, however, also dampen the potential benefits. In the following, the effect of introducing constraints A–C is quantified and analyzed.

### The effect of assignment constraints

In Table 7 we show the effect of imposing constraint A to the collaborative solution.

**Table 7:** The costs of keeping at least $\frac{1}{3}$ and $\frac{2}{3}$ of the initial customers with respect to the collaborative solution without assignment constraints

Instances	n = 30		n = 45		Instances	n = 30		n = 45	
	$\frac{1}{3}$	$\frac{2}{3}$	$\frac{1}{3}$	$\frac{2}{3}$		$\frac{1}{3}$	$\frac{2}{3}$	$\frac{1}{3}$	$\frac{2}{3}$
TSP_O1	6.16	9.16	7.70	8.67	VRP_O1	0.86	1.64	1.36	2.81
TSP_O2	9.21	16.34	12.91	16.23	VRP_O2	1.76	5.05	2.31	7.35
TSP_O3	21.03	31.46	22.49	29.46	VRP_O3	2.95	11.33	3.60	12.13
Average	12.13	18.99	14.37	18.12	Average	1.86	6.01	2.43	7.43

All numbers are reported in percentage points

In the case of MDTSPPD, the restriction that each carrier has to keep at least $\frac{2}{3}$ of their initial customers decreases potential benefits from well over 20% by about 13% to only around 7%. The cost of introducing this constraint is especially high in setting O3, where the highest cost savings were possible.

Even though there is a smaller total collaboration gain for the multi-vehicle case, slightly higher potential savings remain relative to the single-vehicle case. Due to the more or less even customer distribution among carriers, the cost of introducing constraint A is rather low. In fact, requiring each carrier to keep at least $\frac{1}{3}$ of their initial customer base only decreases potential savings by around 2%. The requirement to keep $\frac{2}{3}$ of the initial customer base is less likely to be fulfilled by the collaborative solution and results in higher costs. Still, savings of around 10% on average can be observed. In all cases, the collaborative gain and the cost of introducing the constraint, increase once again with the degree of regional customer overlap.

Table 8 shows the effect of imposing constraint B to the collaborative solution.

**Table 8:** The costs of keeping at least $\frac{1}{3}$ and $\frac{2}{3}$ of the initial number of customers with respect to the collaborative solution without assignment constraints

Instances	n = 30		n = 45		Instances	n = 30		n = 45	
	$\frac{1}{3}$	$\frac{2}{3}$	$\frac{1}{3}$	$\frac{2}{3}$		$\frac{1}{3}$	$\frac{2}{3}$	$\frac{1}{3}$	$\frac{2}{3}$
TSP_O1	2.25	5.33	3.68	6.71	VRP_O1	0.27	1.10	0.44	1.24
TSP_O2	8.19	13.97	10.83	13.53	VRP_O2	0.32	2.81	0.22	2.17
TSP_O3	17.18	27.03	15.50	23.15	VRP_O3	0.40	2.61	0.67	2.46
Average	9.21	15.44	10.01	14.46	Average	0.33	2.17	0.44	1.96

All numbers are reported in percentage points

The requirement to keep at least a given number of customers—regardless of who they were initially assigned to—allows for a more efficient customer allocation to more closely located depots. The cost of introducing this constraint is therefore considerably lower than of constraint A. Again, it can be observed that the level of competition influences the cost of introducing constraint B, as well as the remaining collaborative gain. Particularly in setting O3, high collaborative gains remain, even when requiring each carrier to keep at least $\frac{2}{3}$ of the initial number of customers. A limit on customer share losses can therefore be imposed as a guarantee for all potential participants. At the same time, some participants have the potential to increase their customer share. Again, it can be observed that the cost of introducing this constraint is particularly cheap for MDVRPPD instances. Imposing constraint B does not significantly decrease the potential collaborative gain. High savings of around 15% remain.

Table 9 reports the costs of introducing constraint C.

**Table 9:** We report the costs of keeping at least 80% and 90% of the initial profit with respect to the collaborative solution without assignment constraints. All numbers are reported in percentage points

Instances	n = 30		n = 45		Instances	n = 30		n = 45	
	80%	90%	80%	90%		80%	90%	80%	90%
TSP_O1	8.02	10.85	7.28	10.08	VRP_O1	1.16	3.25	3.11	4.53
TSP_O2	17.75	21.09	15.23	18.16	VRP_O2	3.36	6.75	2.86	4.77
TSP_O3	24.99	31.83	24.56	30.74	VRP_O3	2.80	6.91	2.48	4.69
Average	16.92	21.26	15.69	19.66	Average	2.44	5.64	2.82	4.66

In the case of MDTSPPD, the costs of introducing this constraint are considerably higher than of constraints A and B. This can be explained by the fact that the constraint was set more restrictively to 80% and 90%, rather than 33% and 67%. Requiring each carrier to keep at least 90% of their initial profit reduces potential benefits to 4% on average. Imposing only a minimum profit of 80%, allows for slightly higher gains of around 8%. In a setting with a high degree of customer overlap, these savings can go above 10%. Nevertheless, savings are relatively low when compared to the huge potential reported in Table 5. However, in the multi-vehicle case, the additional costs of imposing an upper bound on profit losses are relatively small. High collaborative gains remain, particularly for competitive settings. Still, effective profit sharing mechanisms as presented by, e.g., Guajardo and Rönnqvist (2016), could be implemented to further increase individual profits.

## Conclusion

The aim of this study was to assess potential trade-offs in collaborative pickup and delivery problems under a centralized collaboration framework. Trade-offs relate to the fact that carriers do not want to share their full set of customers with collaboration partners or want to preserve a minimum share of their pre-collaboration profit. The mathematical formulation and an ALNS solution approach were provided. This approach was applied to publicly available single-vehicle and multi-vehicle test instances.

The computational study revealed that collaboration can lead to potential savings of up to 40%. However, it was shown that these high gains come at the cost of an uneven workload distribution. The numerical results therefore confirmed the concerns of transport companies. Hence, assignment constraints were introduced to generate more acceptable solutions for possible participants and thereby eliminate barriers to entry. The constraints enable carriers to keep at least some of their initial customers or provide upper bounds for profit and customer share losses.

For the single-vehicle case, the introduction of these constraints resulted in considerably increased costs and reduced collaborative gains. However, results revealed that if each carrier operates multiple vehicles, collaboration can provide a high potential for cost savings even when assignment constraints are introduced. Carriers may therefore explicitly exclude certain long-term valued customers from reassignment and still benefit from the collaboration.

We could show that high collaborative gains remain, particularly for competitive settings. Thus, especially last-mile deliveries which are characterized by high competition could therefore profit from horizontal collaborations. Apart from increasing efficiency in this sector, cities could profit from environmental aspects. Due to the complexity of the optimization problem, we limited our research to the deterministic case. The dynamic stochastic setting seems absolutely worth investigating as well. In particular, considering volatile profits due to stochastic travel times might lead to very interesting findings. For this, a discrete-event or agent-based simulation study—or a combination of both—seems recommendable. The proposed solution approach could, for instance, be assessed within a dynamic and stochastic environment in a rolling horizon setting as it is suggested by Sprenger and Mönch (2012) for a real-world scenario in the German food industry. We recommend to run simulations, where carriers, who face stochastic delivery times and dynamically arriving new customer orders, find new assignments of requests to the collaboration on a regular basis. After a given number of periods the proposed solution approach should be used to calculate the new assignments taking newly arrived orders as well as updated information on travel times into account. This would provide valuable managerial insights in how to handle dynamically changing pools of customer orders in a stochastic environment. This is of particular interest in case of carriers insisting on a minimum level of individual collaboration profits, as it was considered in this study.

Hopefully, our findings and ideas will inspire new projects to exploit the benefits of horizontal collaborations.

## Footnotes

1 TSP: http://prolog.univie.ac.at/research/FairInst/Fairness_instances.zip.

2 VRP: http://prolog.univie.ac.at/research/BundlingInst/Instances.zip.
